# Neuronal network maturation differently affects secretory vesicles and mitochondria transport in axons

**DOI:** 10.1038/s41598-018-31759-x

**Published:** 2018-09-07

**Authors:** Eve Moutaux, Wilhelm Christaller, Chiara Scaramuzzino, Aurélie Genoux, Benoit Charlot, Maxime Cazorla, Frédéric Saudou

**Affiliations:** 1 Grenoble Institute of Neuroscience, Université Grenoble Alpes, F-38000 Grenoble, France; 2 INSERM, U1216, F-38000 Grenoble, France; 3 CNRS UMR5214, Institut d’Electronique et des Systèmes, F-34000 Montpellier, France; 4 Université Montpellier 2, F-34000 Montpellier, France; 5 CHU Grenoble Alpes, F-38000 Grenoble, France

## Abstract

Studying intracellular dynamics in neurons is crucial to better understand how brain circuits communicate and adapt to environmental changes. In neurons, axonal secretory vesicles underlie various functions from growth during development to plasticity in the mature brain. Similarly, transport of mitochondria, the power plant of the cell, regulates both axonal development and synaptic homeostasis. However, because of their submicrometric size and rapid velocities, studying the kinetics of these organelles in projecting axons *in vivo* is technically challenging. In parallel, primary neuronal cultures are adapted to study axonal transport but they lack the physiological organization of neuronal networks, which in turn may bias observations. We previously developed a microfluidic platform to reconstruct a physiologically-relevant and functional corticostriatal network *in vitro* that is compatible with high-resolution videorecording of axonal trafficking. Here, using this system we report progressive changes in axonal transport kinetics of both dense core vesicles and mitochondria that correlate with network development and maturation. Interestingly, axonal flow of both types of organelles change in opposite directions, with rates increasing for vesicles and decreasing for mitochondria. Overall, our observations highlight the need for a better spatiotemporal control for the study of intracellular dynamics in order to avoid misinterpretations and improve reproducibility.

## Introduction

Understanding mechanisms that drive the establishment, maturation, function and dysfunction of neuronal networks on a subcellular level requires microscopic approaches that are often technically challenging in the *in vivo* context. The motile nature and submicrometric size of cellular organelles make their study extremely difficult *in vivo* because it requires technology with high spatial and temporal resolution that have yet to be developed. This is particularly true for axonal trafficking of dense core vesicles (DCV) that transports key elements for neuronal growth and transmission. In fact these organelles that are only few hundreds nanometers in size can travel at several micrometers per seconds^1^, which make them extremely difficult to track. Similarly, the relatively small size and highly dynamic nature of mitochondria renders their observation equally challenging *in vivo* and requires high-resolution and high-frequency image acquisitions^2^. Consequently, the exact molecular events controlling subcellular rearrangements and intracellular trafficking in axons and in dendrites within neuronal networks are not fully understood.

One way to overcome these limitations is to use primary cultures of neurons that are extracted from embryonic brain and seeded in a dish. However without a proper control of neurite outgrowth and directionality, neurons often make random, nonspecific, multidirectional and uncontrolled synaptic contacts that may jeopardize the validity of observations. The difficulty to recapitulate the complexity of brain networks composed of multiple neuronal identities complicates the assessment of microscopic events at homo- or heterotopic synapses. In addition, intracellular dynamics are often studied at a unique time point within a given culture, although intracellular dynamics may vary between developing and matured neurons, but also from one culture condition to another. This lack of rigorous temporal identification may therefore affect the dynamicity of organelles and may lead to discrepancies between studies. Therefore, there is a crucial need to develop culture systems that could bridge the gap between *in vivo* and *in vitro* analyses and that would allow systematic and reproducible analyses of intracellular dynamics.

We recently reported an *in vitro* microfluidic system for recording intracellular dynamics with spatial and temporal control by reconstituting a compartmentalized, oriented and functional neuronal network^3^. Space compartmentalization was achieved using a 3-chamber microfluidic design allowing the separation of the different components of neuron’s architecture (soma, dendrites, axon and synapses)^3,4^. Time compartmentalization was achieved by determining the different stages of neuronal network development using selective markers of neurite outgrowth, synapse formation and transmission, as well as neuronal activity. Because of the standardized architecture and specific physical and chemical constraints of the microfluidic platform, neuronal networks develop with specific kinetics that are similar through different devices. In this configuration, network development can be synchronized between different conditions, thus facilitating systematic analyses and reproducibility^5–7^. Using these spatiotemporal features, we cross-compared axonal trafficking of two motile organelles, dense core vesicles and mitochondria, throughout network maturation. We found marked changes in the dynamicity of axonal trafficking for both organelles that correlated with the progressive maturation of the network. Interestingly, trafficking kinetics of vesicles and mitochondria evolved in opposite directions, as demonstrated by the progressive acceleration and densification of anterograde vesicles compared to the dramatic reduction in motile mitochondria in mature axons.

## Results

### Space-time compartmentalization of the corticostriatal network allows the analysis of axonal transport during neuronal network formation

**Figure 1:**
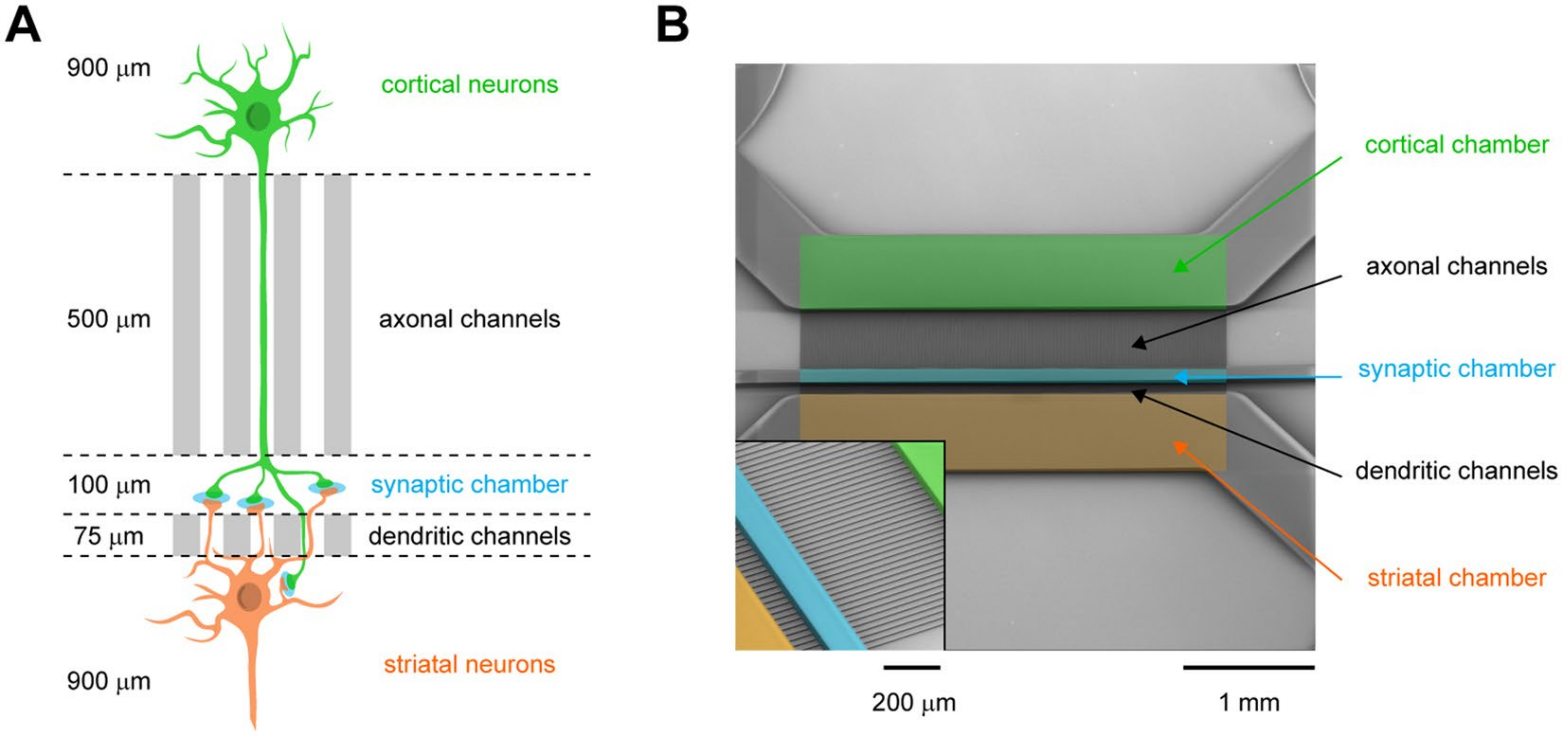
*In vitro* reconstruction of corticostriatal connections using microfluidics. (**A**) Schematic representation of the 3-compartment microfluidic device used for the reconstruction of corticostriatal connections. Cortical neurons (upper chamber, green) connect striatal neurons (lower chamber, orange) via microchannels and an intermediate synaptic chamber (middle chamber, blue). The length of microchannels allows cortical axons (>450 μm) and striatal dendrites (<450 μm) to reach and connect in the synaptic chamber^3,4^. (**B**) Microphotograph of the silicium template used for the fabrication of PDMS-based microfluidics. The different compartments are reported.

We have recently developed a microfluidic-based approach that enables the reconstruction of a time- and space-controlled neuronal network compatible with fast spinning confocal videomicroscopy^3^. This system uses a silicon polymer-based microfluidic device composed of two fluidically-isolated neuronal chambers that are connected via a set of thin microchannels through which neurites can grow and contact each other in an intermediate synaptic compartment. We previously determined optimal conditions using physical and chemical constraints (*i*.*e*. dimensions and coating substrates, respectively) so that neuronal cultures between distinct devices are standardized and can be compared^3^. Using primary neuron cultures, we reconstituted a corticostriatal network in which cortical neurons project to striatal target neurons through oriented axodendritic connections (Fig. 1A,B). Because of the spatiotemporal feature of the platform, each cellular compartment of the neuron (*i*.*e*. soma, dendrites, axons and synapses) can be systematically identified at each stage of the culture, which allows to establish a temporal map of intracellular dynamics. We therefore took advantage of this system to cross-compare the trafficking kinetics of axonal DCVs and mitochondria at different stages of network maturation [4, 7, 10, 14 and 21 days *in vitro* (DIV)].

**Figure 2:**
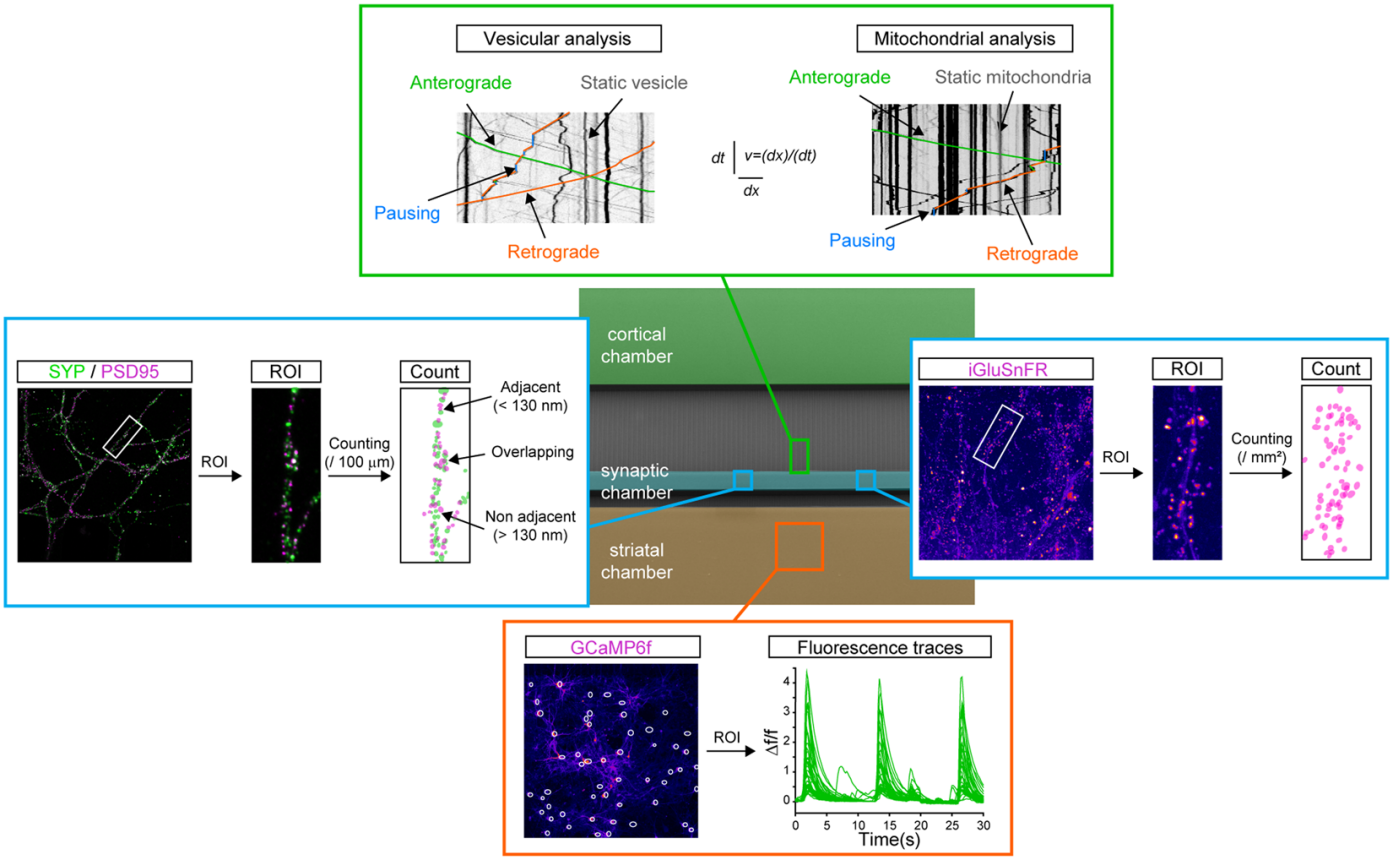
Experimental setup for the concomitant recording of axonal trafficking and network maturation. Transport dynamics of dense core vesicles and mitochondria (upper green box) were recorded in the distal part of axonal channels and kymographs were analyzed using the indicated kinetic parameters. Synaptic formation (left blue box) was analyzed by quantifying the number of adjacent spots of synaptophysin (presynaptic) and PSD95 (postsynaptic) per 100 μm. Glutamatergic transmission (right blue box) was assessed by quantifying the number of iGluSnFR spots per mm^2^ after cortical stimulation (right). Network activity and synchrony (lower orange box) was assessed using calcium imaging of GCaMP6f in striatal neurons.

To study the dynamics of DCVs, we used lentiviral expression of Brain-Derived Neurotrophic Factor fused to the fluorescent mCherry protein (BDNF-mCh) in cortical neurons. BDNF is a prototypical cargo of DCVs endogenously expressed in cortical neurons and transported in axons to promote the survival of striatal neurons^8,9^. Mitochondrial transport was analyzed using cytochrome c oxidase subunit VIII fused to the fluorescent DsRed2 protein (Mito-DsRed2), a widely used reporter of mitochondria trafficking^10^. Videorecording was performed at high frequency and high resolution using spinning-disk confocal videomicroscopy. To ensure that organelle trafficking was recorded in axons only, the field of acquisition was placed over the most distal section of the 500 µm-long channels (Fig. 2, green box), since axons but not dendrites are able to grow to such lengths^11^. Kinetic parameters were then extracted from kymograph analyses using the KymoToolBox plugin in ImageJ^10^. Trafficking was categorized as anterograde (soma to synapse), retrograde (synapse to soma), pausing (<0.12 μm/s for DCVs; <0.02 μm/s for mitochondria) and static (no movement). The velocity and number of motile units were used to evaluate the net directional flux (which gives indications about the global directionality of the flux) and the linear flow rate (which represents the global amount of material moving).

In parallel, different markers of neuronal maturation were used to determine the temporal organization of the network. First, synapse formation was estimated using presynaptic and postsynaptic markers, respectively synaptophysin (SYP) and postsynaptic density protein-95 (PSD95) (Fig. 2, left blue box). Immunostaining of both markers was performed in the intermediate synaptic chamber and Airyscan-treated confocal images were analyzed to determine the number of adjacent (<130 nm) SYP/PSD95 spots per 100 μm neurites. We previously reported that PSD95 immunostaining colocalizes with striatal but not cortical branches and that the synaptic chamber is enriched in cortical axons and striatal postsynaptic dendrites, indicating the formation of oriented axodendritic corticostriatal synapses^3^. Corticostriatal transmission was determined using the glutamate sensor iGluSnFR^12^ that selectively measures glutamate release from cortical axons since cortical projecting neurons are glutamatergic while striatal neurons are GABAergic^13,14^ (Fig. 2, right blue box). Striatal neurons were therefore infected with lentiviruses expressing iGluSnFR and the number of fluorescent spots on striatal dendrites was counted in the synaptic chamber after chemical stimulation of cortical neurons. Finally, corticostriatal network connectivity, an important aspect of network maturation, was analyzed using the genetically-encoded calcium indicator GCaMP6f^15^ expressed in striatal neurons (Fig. 2, lower orange box). Spontaneous, synchronous activity was analyzed by calcium imaging in the striatal compartment.

### Progressive formation and maturation of corticostriatal networks in the microfluidics

**Figure 3:**
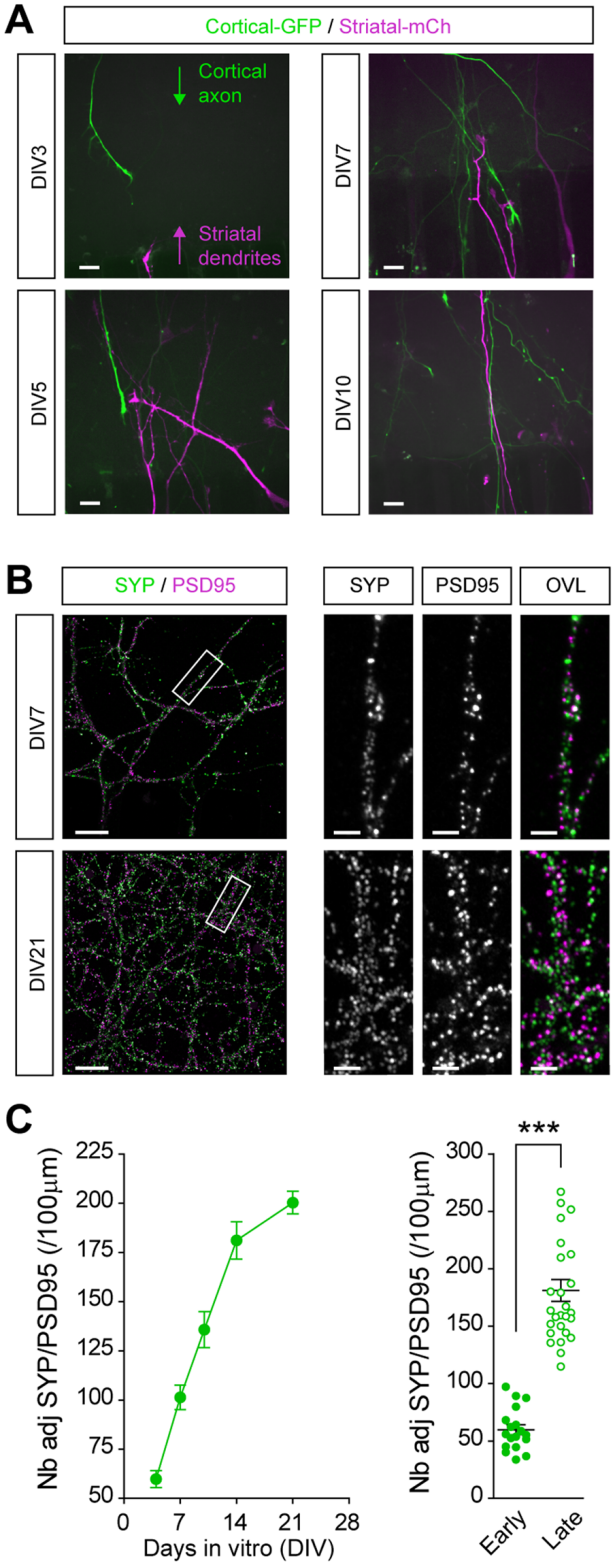
Time-course of corticostriatal network formation. (**A**) Time recording of neurite outgrowth in the synaptic chamber using lentiviral expression of GFP and mCherry in cortical and striatal neurons, respectively. Images show the growth of cortical axons and striatal dendrites in the synaptic chamber from 3 to 10 days *in vitro* (DIV). Scale bars, 10 μm. (**B**,**C**) Time recording of the formation of corticostriatal connections in the synaptic chamber using selective markers for the presynaptic (synaptophysin, SYP) and postsynaptic (PSD95) compartments. (**B**) Images show SYP/PSD95 colocalization at DIV 7 and DIV 21. Insets show a 10x magnification of boxes in the left panel. Scale bars, 10 μm (insets, 2 μm). (**C**) Quantification of the number of SYP/PSD95 adjacent spots per 100 μm branches shows the progressive formation of synaptic contacts until DIV 14 (F_4,112_ = 58.3, p < 0.0001; n = 23) and stabilization afterwards (DIV 14 vs DIV 21, n.s). The bar graph recapitulates data obtained at early (DIV 4) and late (DIV 14) stages of neuronal culture. ***p < 0.0001.

To establish a temporal map of network formation we first recorded cortical and striatal neurite outgrowth in the synaptic chamber. We previously reported that the different lengths of microchannels combined with a gradient of poly-*D*-lysine/laminin coating in the microfluidics enriches the synaptic chamber in cortical axons and in striatal dendrites while preventing non-physiological striato-cortical connections^3^. We found that GFP-labeled cortical axons and mCh-labeled striatal dendrites reached the synaptic chamber as soon as DIV 3 (Fig. 3A). Around DIV 5, cortical axons started making contacts with striatal dendrites in the synaptic chamber and continued branching until DIV 10. We next recorded the progression of synapses formation using AiryScan confocal images of presynaptic synaptophysin and postsynaptic PSD95 in the synaptic chamber (Fig. 3B,C). We found that the first synaptic contacts formed at DIV 4, although their morphology indicated large, immature clusters of presynaptic and postsynaptic elements. Starting from DIV 7, the morphology of synapses progressively changed, switching from sparse and large clusters to dense and small juxtaposed spots. The number of contacts also steadily increased until reaching a plateau between DIV 14 and 21.

**Figure 4:**
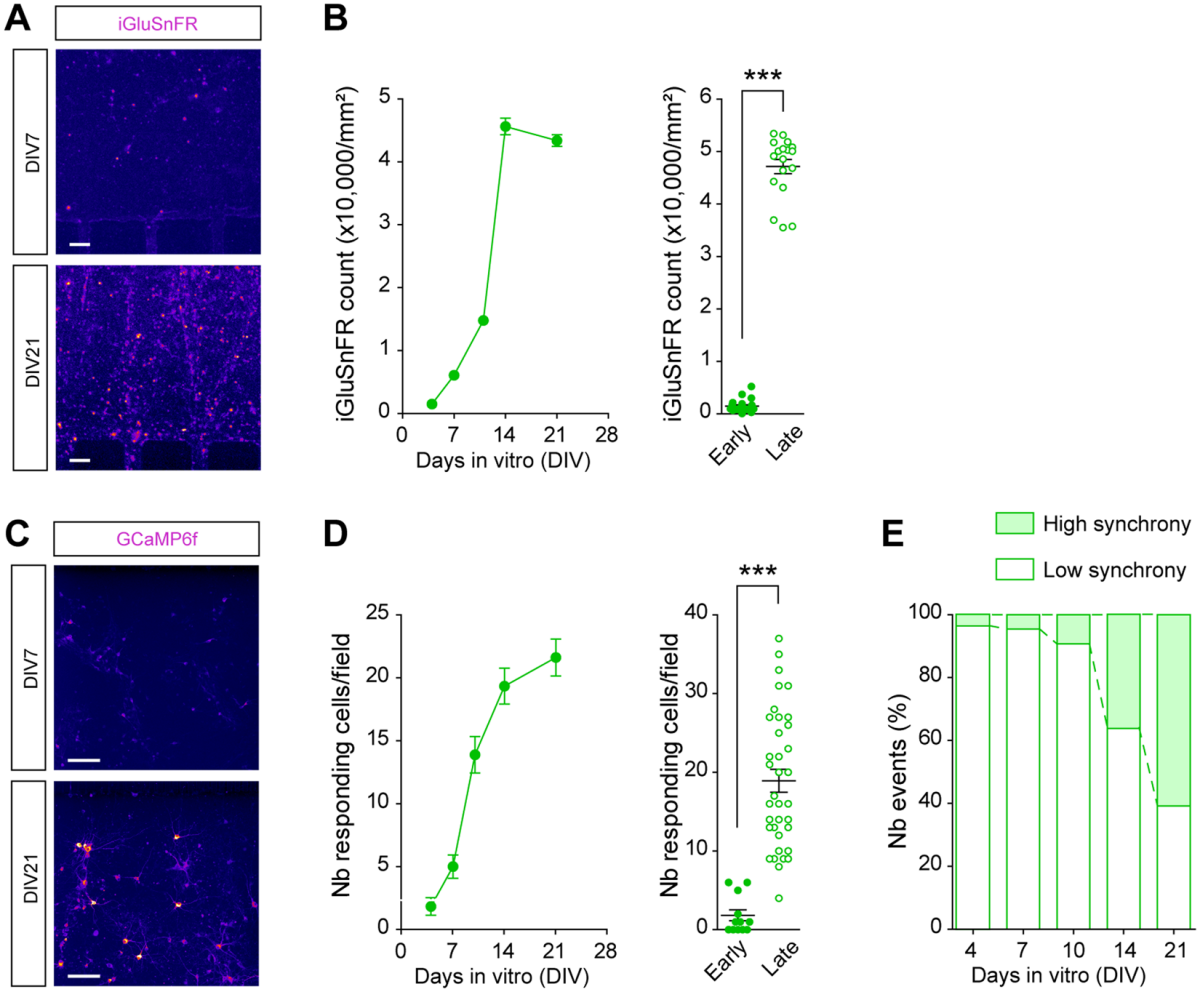
Time-course of corticostriatal network activity and maturation. (**A**,**B**) Time recording of corticostriatal excitatory transmission in the synaptic chamber using the glutamate sensor iGluSnFR. (**A**) Images show iGluSnFR fluorescence after chemical stimulation of cortical neurons at DIV 7 and DIV 21. Spots indicate the presence of active corticostriatal synaptic transmission. Scale bars, 10 μm. (**B**) Quantification of the number of iGluSnFR spots per mm^2^ shows progressive maturation of synapse transmission from DIV 4 to DIV 14 (F_4,126_ = 566.3, *p* < 0.0001; n = 26) and stabilization afterwards (DIV 14 *vs* DIV 21, n.s). The bar graph recapitulates data obtained at early (DIV 4) and late (DIV 14) stages of neuronal culture. ***p < 0.0001. (**C-E**) Time recording of network connectivity in the synaptic chamber using the Calcium indicator GCaMP6f. (**C**) Images show a stack image obtained from a 30-sec video of spontaneous GCaMP6f activity at DIV 7 and DIV 21 (see Movies S1 and S2). Scale bars, 100 μm. (**D**) Quantification of the number of cells with GCaMP6f activity shows the progressive recruitment of active neurons in the network from DIV 4 to DIV 14 (F_4,121_ = 32.80, *p* < 0.0001; n = 25 fields) and their stabilization afterwards (DIV 14 *vs* DIV 21, n.s). The bar graph recapitulates data obtained at early (DIV 4) and late (DIV 14) stages of neuronal culture. ***p < 0.0001. (**E**) Distribution of the number of events shows the progression of synchronized events from low synchrony (<50% synchrony between events) at DIV 4 to high synchrony (>50%) at DIV 21.

We next established a temporal map of functional maturation by recording glutamate corticostriatal transmission and calcium imaging overtime (Fig. 4). Using the iGluSnFR sensor^12^, we found almost no functional transmission in the early stages of network formation (DIV 4–7). The number of iGluSnFR-positive spots then progressively increased until reaching a steady state at DIV 14, indicating that the network was fully functional after 2 weeks in culture (Fig. 4A,B). Similar kinetics were obtained using GCaMP6f imaging. Limited activity was detected before the formation of synaptic contacts (DIV 7), and then the number of responding cells constantly increased until reaching a plateau at DIV 14 (Fig. 4C,D and Movies S1 and S2). In addition, spiking activity reorganized from sparse and random bouts of events to frequent and synchronized bursts, as shown by the increased proportion of synchronous events in the later stages of culture (Fig. 4E).

**Figure 5:**
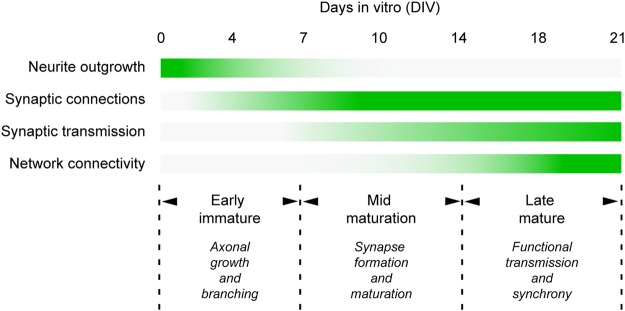
Summary of network formation and maturation in microfluidics. In the early stage of the culture (DIV 0 to 7, Early), cortical axons and striatal dendrites grow and branch into the synaptic chamber where they form immature contacts. Then (DIV 7 to 14, Mid), synapses form and progressively mature to establish functional excitatory connections that transmit information from cortical to striatal neurons. In the late stage (DIV 14 to 21 and beyond, Late), the system is fully functional with spontaneous and synchronized communication between cortical and striatal neurons.

Together, these observations indicate that the microfluidic device recapitulates a functional network *on-a-chip* with a spatial and temporal identification of each stage of maturation (Fig. 5). By combining these results altogether, we could defined three main stages of network maturity: The early immature stage (DIV 1–7), which corresponds to the growth of cortical axons and striatal dendrites through their respective microchannels (Fig. 3A) and to the early formation of non-functional synaptic contacts (Figs 3C and 4B); The mid maturation stage (DIV 7–14), which corresponds to an increase in axonal branching and synapses (Fig. 3A,C) that progressively become functional (Fig. 4); The late mature stage (>DIV 14), which corresponds to a fully functional, stable corticostriatal network in which synaptic transmission and neuronal activity have reached an optimal state (Fig. 4B,D and E).

### Vesicular motility increases with neuronal network maturation

**Figure 6:**
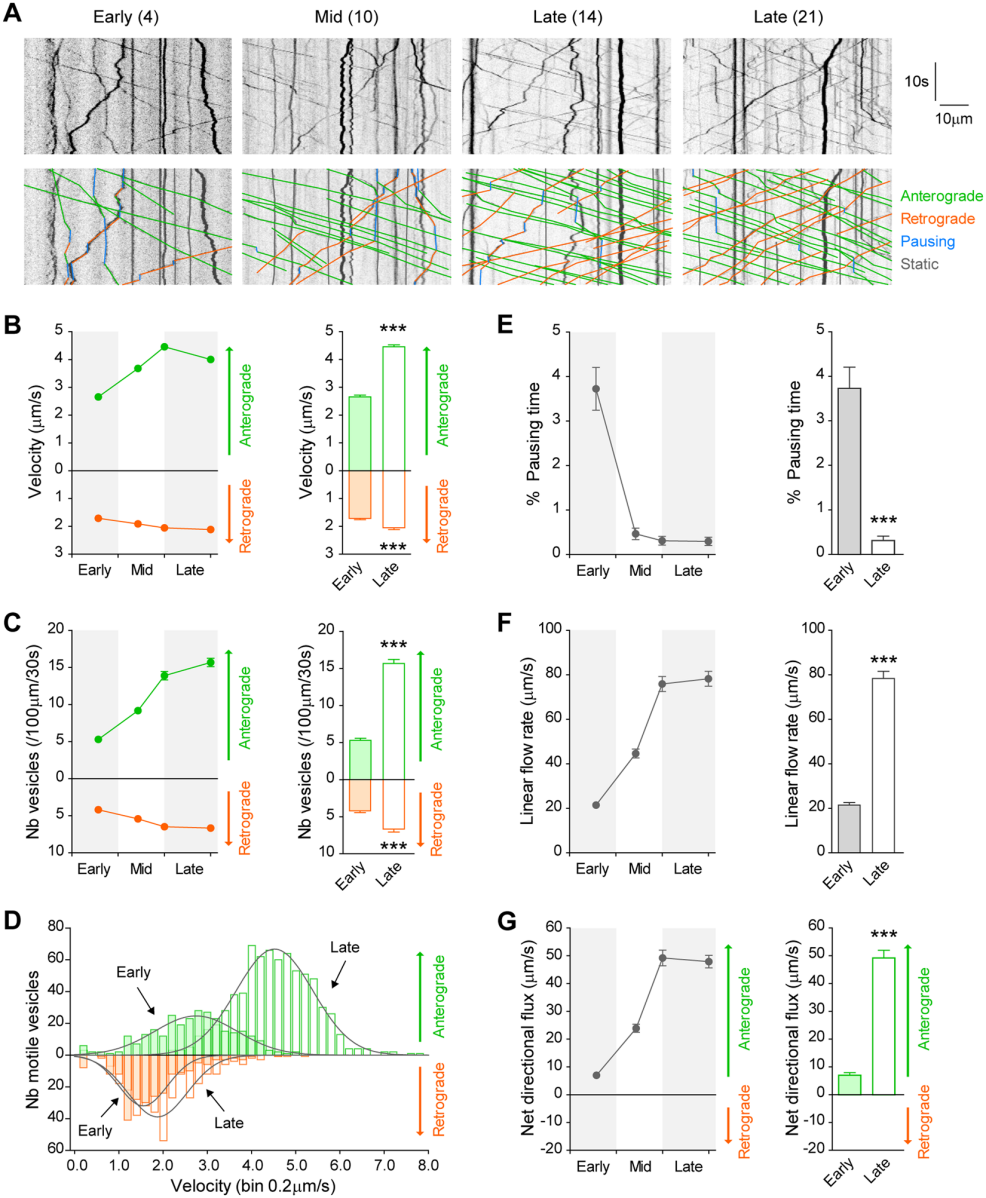
Progressive acceleration of axonal vesicular flow during network maturation. (**A**) Kymograph analyses obtained at different stages of network maturation (early, mid, late) show progressive increase in axonal transport of dense core vesicles. Number in brackets indicates DIV. Anterograde and retrograde movements, pauses and static vesicles are shown. (**B**) Increased segmental velocity (Antero, F_3,201_ = 121.3, *p* < 0.0001; Retro, F_3,201_ = 11.5, *p* < 0.0001; n = 52) and (**C**) increased number of vesicles (Antero, F_3,201_ = 105.9, *p* < 0.0001; Retro, F_3,201_ = 14.7, *p* < 0.0001) translate into (**D**) a redistribution of the pool of motile vesicles (Antero, Χ^2^ = 452.8, *p* < 0.0001; Retro, Χ^2^ = 26.26, *p* < 0.0001). (**E**) The pausing time of motile vesicles rapidly decreases to a minimum at mid stages (F_3,201_ = 42.1, *p* < 0.0001). These kinetics result in (**F**) increased global linear flow rate (F_3,201_ = 109.5, *p* < 0.0001) and (**G**) increased anterograde net flux (F_3,201_ = 103.0, *p* < 0.0001) of axonal vesicles that stabilize after DIV 14 (DIV 14 *vs* DIV 21, n.s). All bar graphs recapitulate data obtained at early (DIV 4) and late (DIV 21) stages of neuronal culture. ***p < 0.0001.

We next studied axonal transport of vesicular organelles by videotracking BDNF-mCh-containing DCVs at each stage of network maturation: early (DIV 4), mid (DIV 10) and late (DIV 14 and DIV 21) (Fig. 6A). Velocity analysis revealed a progressive acceleration of both anterograde and retrograde DCVs in axons until reaching a plateau at 4.46 ± 0.07 μm/s (anterograde) and 2.05 ± 0.06 μm/s (retrograde) at DIV 14 when the network is fully mature (Fig. 6B). The number of motile DCVs also increased by a factor 3 (anterograde) and 1.5 (retrograde) from early to late stages of network maturation (Fig. 6C). Interestingly, retrograde and anterograde transport did not evolve in the same manner over the course of maturation. Retrograde kinetics remained fairly similar throughout each stage of development, whereas anterograde transport became progressively denser, with high motility and fast velocities (3 to 7 μm/s) when the network is mature compared to few motile events and slow velocities (1 to 4 μm/s) in the early stage (Fig. 6D). In addition, moving vesicles demonstrated shorter pausing periods during movement over time, leading to increased processivity of motile vesicles (Fig. 6E). Together, these changes led to a progressive 4-fold increase in the linear flow rate of traveling secretory vesicles within the axon, with a preferential anterograde flux toward the synapse that also became significantly more pronounced (5-fold increase) with maturation (Fig. 6F,G).

### Mitochondrial dynamics decreases with network maturation

**Figure 7:**
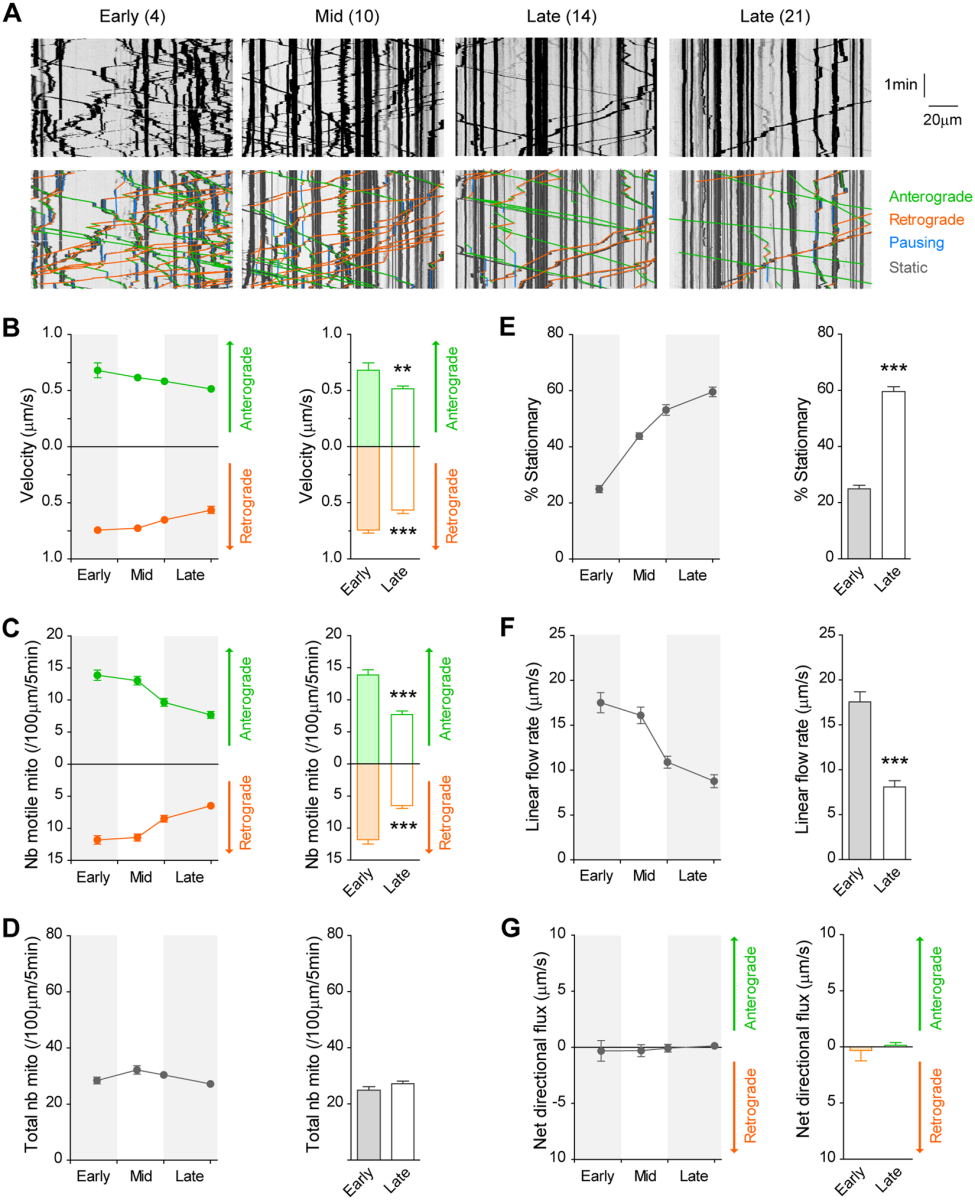
Progressive reduction in axonal transport of mitochondria during network maturation. (**A**) Kymograph analyses at early, mid and late stages of network maturation show a progressive decrease in mitochondria motility. Number in brackets indicates DIV. Anterograde and retrograde movements, pauses and stationary mitochondria are shown. (**B**) Decreased segmental velocity (Antero, F_3,225_ = 3.1, *p* < 0.05; Retro, F_3,225_ = 10.0, *p* < 0.0001; n = 57) and (**C**) decreased number of motile mitochondria (Antero, F_3,225_ = 15.9, *p* < 0.0001; Retro, F_3,225_ = 18.9, *p* < 0.0001). (**D**) The total number of mitochondria remains constant over time. (**E**) Increased percentage of stationary mitochondria (F_3,225_ = 98.9, *p* < 0.0001). (**F**) Decreased velocity and number of mitochondria lead to a global decrease in linear flow rate (F_3,225_ = 25.7, *p* < 0.0001) until DIV 14 (DIV 14 *vs* DIV 21, n.s) but (**G**) no directional preference could be observed. All bar graphs recapitulate data obtained at early (DIV 4) and late (DIV 21) stages of neuronal culture. ***p < 0.01, ***p < 0.0001.

We then studied mitochondrial trafficking in cortical axons from early to late stages of development using the Mito-DsRed2 mitochondrial marker (Fig. 7A). We observed a progressive decrease in both anterograde and retrograde velocities that were accompanied by a net 2-fold decrease in the number of motile mitochondria in both directions between DIV 4 and DIV 21 (Fig. 7B,C). It must be noted that mitochondrial top speeds (~0.7 μm/s) were much lower than those of DCVs (~4.5 μm/s) and the number of motile mitochondria (~1.5 events/100 μm/30 s) was 10 times lower than that of vesicles (~15 events/100 μm/30 s). The progressive changes in the number of motile units were also comparable between DCVs and mitochondria, although in opposite direction (3- and 1.5-fold increase in the number of anterograde and retrograde BDNF vesicles compared to 2-fold decrease for motile mitochondria). In addition, because the total number of mitochondria remained constant over time (Fig. 7D) the decrease in motile units translated into an increased proportion of stationary mitochondria in the axon (Fig. 7E). The combination of decreased mobility and velocities further led to a global decrease in the linear flow rate upon network maturation (Fig. 7F). In contrast to DCVs trafficking, we did not observe any preferential directionality of mitochondria flux during formation or maturation of the network (Fig. 7G).

We verified these results using another non-related mitochondria chemical reporter, Mitotracker^16^. To do so, cortical neurons were transiently incubated with the dye at DIV 4 (early immature) or at DIV 14 (late mature) and mitochondria axonal trafficking was analyzed (Supplementary Fig. S1). We confirmed the previously observed decrease in motility and dynamicity of axonal mitochondria as the system matures, suggesting that these changes are not influenced by the nature of the reporter but are rather due to cellular regulations.

### DCV and mitochondria axonal trafficking are oppositely regulated during neuronal network maturation

**Figure 8:**
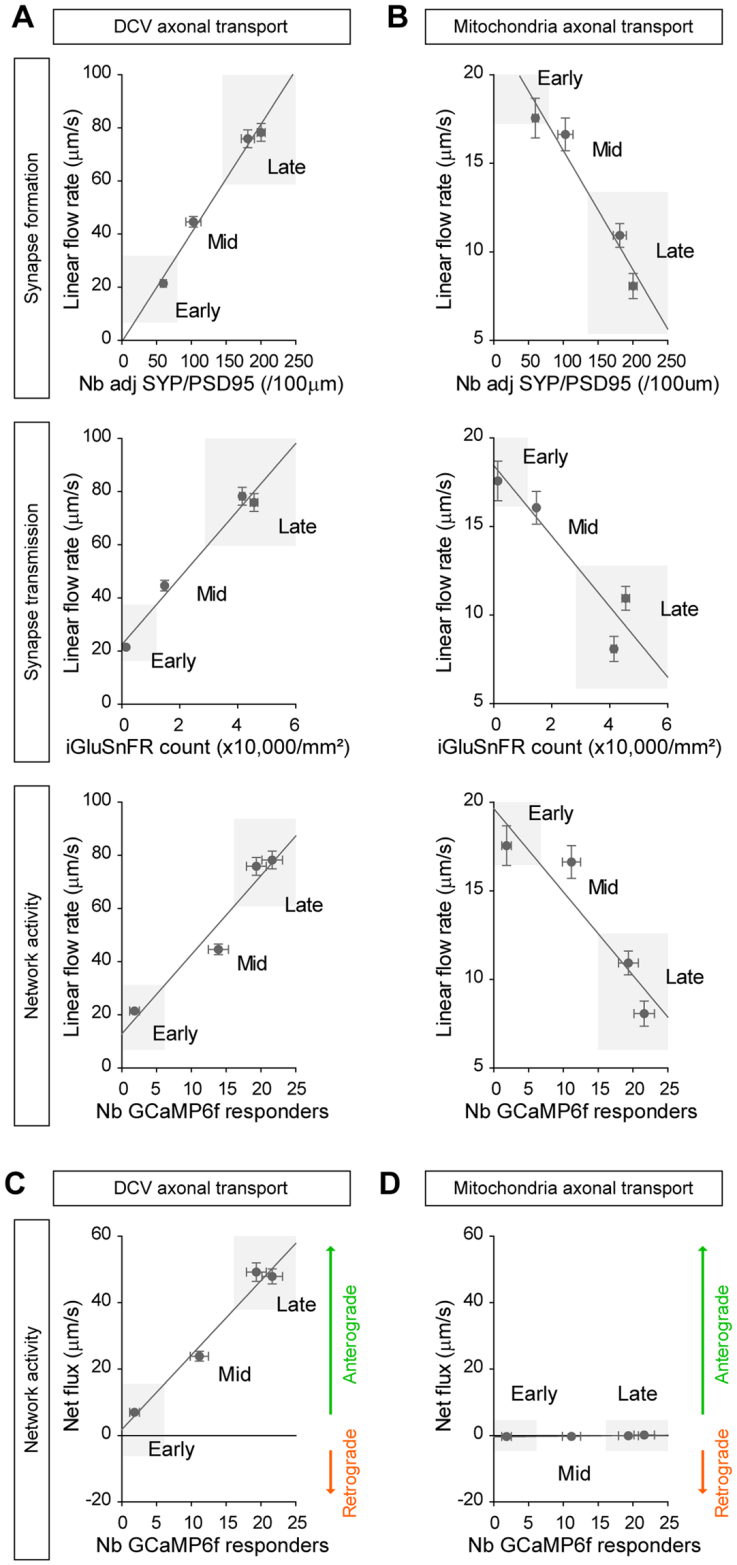
Opposite correlations between vesicular and mitochondrial trafficking with network maturity index. (**A**) DCV axonal transport is positively correlated with network morphological formation (SYP/PSD95, R^2^ = 0.61, *p* < 0.0001), network functional maturation (iGluSnFR, R^2^ = 0.61, *p* < 0.0001) and global network activity (GCaMP6f, R^2^ = 0.58, *p* < 0.0001). (**B**) Conversely, mitochondria axonal trafficking is negatively correlated with network formation (SYP/PSD95, R^2^ = 0.95, *p* < 0.05), functional maturation (iGluSnFR, R^2^ = 0.86, *p* < 0.05) and global activity (GCaMP6f, R^2^ = 0.86, *p* < 0.05). Correlations were obtained by using the linear flow rate of DCV and mitochondria as a representative readout of trafficking, and synapse formation (number of adjacent SYP/PSD95 spots from Fig. 3C), glutamate transmission (iGluSnFR counts from Fig. 4B) and network activity (number of GCaMP6f responding cells from Fig. 4D) to determine a network maturity index. (**C**) The net directional flux of DCVs is also positively correlated with network maturity (R^2^ = 0.59, *p* < 0.0001), highlighting the switch from balanced to high anterograde transport as the network matures. (**D**) In contrast, mitochondria show no directional preferences overtime (R^2^ = 0.78, n.s).

Vesicular and mitochondrial transport progressively changed with neuronal network development, but did so in opposite manners. Because microfluidics allow the spatial and temporal identification of neuronal compartments we could correlate the evolution of intracellular trafficking with each stage of neuronal maturation (Fig. 8). We first determined an index of network maturity by using three independent measurements of network maturation: synapse morphology (number of adjacent synaptophysin and PSD95 spots), synapse function (number of iGluSnFR-positive spots) and global neuronal activity (number of GCaMP6f-responding cells). We next used the linear flow rate as a representative readout of axonal trafficking for both DCVs and mitochondria because it takes into account the number and velocity of moving organelles, the two main sites of regulation of trafficking. Using this correlative analysis, we found that the linear flow rate of secretory vesicles positively correlated with maturation, indicating that axonal transport becomes faster and more intense as the system matures. Similar effects were found with all three parameters (Fig. 8A).These changes in the number and dynamicity of DCVs upon maturation of neuronal network, as well as their preferential anterograde directionality, are in line with the need for new membranes and cargoes during axonal growth and synapse maturation^17^. Conversely, the linear flow rate of mitochondria negatively correlated with network maturity (Fig. 8B), demonstrating a progressive reduction in mitochondrial transport during maturation. This decrease in both velocity and number of motile mitochondria upon maturation in our reconstructed neuronal network agrees with recent studies reporting decreased motility of mitochondria during development of cortico-cortical projecting neurons *in vivo*^2^.

We then compared the net flux of DCVs and mitochondria (*i.e*. their directionality) with global neuronal activity (Fig. 8C,D). The net flux of axonal DCVs was positively correlated with maturation, indicating that the flux of secretory vesicles becomes progressively more anterograde in the late stages of the culture. This tight correlation between anterograde transport of axonal vesicles and neuronal activity is in accordance with increased synaptic release during high neuronal activity, especially for BDNF-containing secretory vesicles^18^. In contrast, despite increased mitochondrial flow in axons there was no correlation between the net flux and network maturation, indicating that both anterograde and retrograde transports changed with the same pace leading to a balanced flux.

Together these observations highlight the need for a better temporal control of the neuronal environment to conclude from changes in intracellular dynamics. They also validate the neuronal network-on-a-chip approach as an efficient system for investigating cellular mechanisms in physiologically relevant systems.

## Discussion

In this study, we used a microfluidic platform that allows the *in vitro* reconstruction of neuronal networks to study intracellular dynamics as a function of neuronal maturation. Because the standardized architecture of the platform allows space and time compartmentalization of neuronal networks, we identified the different stages of network maturation using systematic analyses with selective markers and live reporters (SYP/PSD95, iGluSnFR, GCaMP6f). By recording axonal trafficking dynamics of two distinct cargoes (DCVs and mitochondria) at different developmental stages of the network, we found opposite changes in their dynamicity that we could directly link with the progressive maturation of the neuronal circuit.

*In vivo*, brain structures such as the cortex and the striatum are physically separated. They connect over long distances via oriented, unidirectional axodendritic contacts. Microfluidic devices are unique tools to isolate and compartmentalize cells *in vitro*. By using an optimized version of the previous generation of compartmentalized microfluidics we reconstructed an *in vitro* corticostriatal network compatible with subcellular investigations^3,4^. In this system, cortical neurons specifically project their axons onto striatal neurons through a series of individual microchannels while cell bodies remain in their respective seeding chambers. This facilitates the study of specific intracellular events in fluidically isolated cell compartments (soma, dendrites, axons). Furthermore, the presence of an intermediate chamber connecting axonal and dendritic channels further isolates synaptic contacts which allows the study of molecular dynamics in pre- and post-synaptic elements of the synapse.

Microfluidics are highly versatile in nature so that they can be used with virtually any types of cells, including neuronal and non-neuronal cells, as long as their primary culture is made possible. Consequently many types of physiological neural networks can be reconstituted *in vitro*, such as cortico-cortical, cortico-hippocampal, hippocampo-hippocampal networks or even neuron-muscle connections^19^. In addition, this system can be used in normal and pathological conditions using neurons from transgenic mouse models^3^ or induced stem cells from human patients, to study cellular and molecular alterations underlying network dysfunctions.

Because microfluidic systems are compatible with high-resolution microscopy they allow the simultaneous analysis of multiple subcellular events, including intracellular transport, synapse dynamics, transmission, neuronal activity and global network synchrony (Fig. 1). In this study we took advantage of the platform to record axonal trafficking while monitoring network formation and maturation. To do so, we used specific fluorescent cargoes to track the transport of secretory DCVs (BDNF-mCh) and mitochondria (Mito-DsRed2) combined with live fluorescent reporters such as GCaMP6f to image calcium dynamics (a readout of neuronal activity)^15^ or iGluSnFR to quantify excitatory transmission (a readout of synapse maturation)^12^. A wide variety of other biological analyses can be envisaged such as nuclear gene expression using luciferase reporter gene^20^, axonal endoplasmic reticulum dynamics using ER-GCaMP-150^21^, activity-dependent cytoskeleton remodeling using live actin imaging^22^, and exocytosis using pHluorin-tagged reporters^23^.

The corticostriatal neuronal circuit is particularly relevant for the study of BDNF trafficking because the trophic support of striatal neurons depends on cortical axons^9^. This is particularly exemplified in corticostriatal disorders such as Huntington’s disease in which BDNF axonal transport is deeply affected^24^. In addition, both BDNF and mitochondria axonal trafficking have been shown to be critical for the development and branching of cortical projecting neurons^25,26^. The combined analyses of BDNF and mitochondria axonal transport using our time-controlled microfluidic device revealed opposite regulatory changes during the development of the system. These observations could be used to further investigate *in vivo* the exact contributions of both cellular event in the growth and branching of cortical axons.

Using the microfluidic platform, we observed that the axonal trafficking of secretory vesicles and mitochondria progressively evolve in opposite directions during network maturation. Our observation of decreased mitochondrial dynamicity agrees with a recent *in vivo* study reporting decreased motility of mitochondria in adult brains compared to developing neuronal circuits^2,27^, which supports the physiological integrity of our reconstituted network. Interestingly, the number of motile mitochondria dramatically decreases in the late, mature stages of network maturation whereas the total number of axonal mitochondria remains stable over time. This 2-fold reduction in motility leads to an increase of stationary mitochondria in the axon over time. In contrast, transport kinetics of DCVs demonstrated increased dynamics upon network maturation. These opposite changes therefore suggest distinct regulatory mechanisms and molecular actors which may be selective for each organelle. In the case of mitochondria trafficking, increased electrical activity and Na^+^ channel activation have been shown to slow axonal transport and to increase the number of stationary mitochondria^28^. This activity-dependent regulation of mitochondrial transport in axons may be mediated by the direct coupling between kinesin and syntaphilin^29^. Immobilization of mitochondria via syntaphilin overexpression has been associated with increased axonal branching and synaptic formation in the distal axon^26^. In contrast, activity-dependent increase in vesicular trafficking has been associated with microtubule polyglutamylation^30^, kinesin phosphorylation^31^ or motor redistribution^32^, although these mechanisms have not been studied specifically in axons and remained to be elucidated. These distinct molecular mechanisms may therefore underlie the opposite changes in mitochondria and vesicular motility during axonal maturation and synapse formation.

In the late, mature stage of the network we observed vesicular velocities ranging from 3 to 7 μm/s, which is much higher than velocities traditionally reported in the literature^10,33,34^. The 1–3 μm/s velocity range recorded in the early, immature stage of network culture is much more comparable to what was found in these studies. Interestingly, changes in vesicular dynamicity are not restricted to velocities. Indeed, the number and directionality of vesicles along the axon both drastically increased throughout network maturation. As a result, the directional flux of axonal vesicles becomes progressively more anterograde toward presynaptic sites which correlates well with the increasing need in synaptic vesicles and secretory proteins at mature synapses^17^. Interestingly, the species used for neuronal culture is also critical for transport kinetics. We previously reported axonal trafficking of DCVs and mitochondria at similar time points using mouse cortical neurons that showed slight differences with the present study using rat cortical neurons^3^. While BDNF axonal trafficking remained quite similar between both studies (see Fig. 2 in^3^), mitochondria axonal transport showed marked differences, especially on the number of motile units (2-fold increase in rat cultures) and linear flow rate (3-fold increase). These observations show that the stage of neuronal maturity as well as the species used for neuronal cultures are important factors of variability that should be taken into account for the reproducibility of observations between studies.

Taken together, our results demonstrate that studying intracellular dynamics in organized and controlled co-cultures, instead of mixed, free neuronal cultures, is crucial to understand molecular mechanisms that occur in mature circuits. These mechanisms are likely to be different for developing versus mature neurons, and will vary depending on the cargo of interest. Furthermore our data reveals the importance of choosing the correct time window for observing biological mechanisms depending on the scientific question in order to improve the reproducibility of studies.

## Materials and Methods

### Primary neuronal culture in microfluidic chambers

Microfluidic chambers were fabricated and prepared as previously described^3^. Microchambers were coated with poly-D-lysin (0.1 mg/ml) in the upper and synaptic chambers, and with a mix of poly-D-lysin (0.1 mg/ml) + laminin (10 µg/ml) in the lower chamber overnight at 4 °C, followed by careful washing with growing medium (Neurobasal medium supplemented with 2% B27, 2 mM Glutamax, and 1% penicillin/streptomycin). Primary cortical and striatal neurons were prepared as previously described^35^. Briefly, cortex and ganglionic eminences were dissected from e17.5 rat embryos, digested with a papain and cysteine solution followed by two incubations with trypsin inhibitor solutions, and gentle mechanic dissociation. Dissociated cortical and striatal neurons were re-suspended in growing medium and plated at a final density of ~7000 cells/mm^2^. Cortical neurons were plated on the upper chamber after addition of growing medium in the synaptic chamber to balance compartmental pressure. Striatal neurons were then added in the lower chamber. Neurons were left in the incubator for at least 3 hours before all compartments were gently filled with growing medium. Fluorescent markers and cargoes were expressed in neurons using lentiviruses (LV) or adeno-associated viruses (AAV) infections at DIV 2, or were electroporated with plasmids using Amaxa Nucleofactor (Lonza) before plating. Cultures in the microfluidics were visually inspected before acquisitions. Low-quality cultures (i.e. containing dead/clustered cells or with cells invading the synaptic chamber) were removed. All experimental procedures were performed in an authorized establishment (Grenoble Institut des Neurosciences, INSERM U1216, license B3851610008) in strict accordance with the recommendations of the European Community (86/609/EEC) and the French National Committee (2010/63) for care and use of laboratory animals.

### Constructs and viruses

The following constructs, LVs and AAVs were used: LV.PGK.GFP (pRRLSIN.cPPT.PGK-GFP.WPRE plasmid #12252 from Addgene), LV.CMV.mCherry (pLV-mCherry plasmid #36084 from Addgene), LV.PGK.iGluSnFR (pCMV(MinDis).iGluSnFR plasmid #41732 from Addgene cloned into pRRLSIN.cPPT.PGK-GFP.WPRE plasmid #12252 by replacing GFP with iGluSnFR), AAV5.SYN.GCaMP6f (#AV-5-PV2822 from U Penn Vector Core facility), LV.PGK.BDNF-mCh^36^ and pDsRed2-Mito (plasmid #632421 from Clontech).

### Mitotracker labeling

Culture medium of cortical neurons was complemented at DIV 3 or DIV 13 with 100 nM Mitotracker-Red CMXRos (Life Technologies, #M7512) for 45 min and then washed twice with fresh NB-B27. Videorecordings were performed 24 h later at DIV 4 or DIV 14, respectively.

### Live-cell and confocal imaging

Live-cell videorecordings were performed using an inverted microscope (Axio Observer, Zeiss) coupled to a spinning-disk confocal system (CSU-W1-T3, Yokogawa) connected to wide-field electron-multiplying CCD camera (ProEM^+^ 1024, Princeton Instrument) and maintained at 37 °C and 5% CO_2_. Images were taken at 5 Hz for 30 s for BDNF-mCh trafficking using a ×63 oil-immersion objective (1.46 NA), at 1 Hz for 5 min for Mito-DsRed2 using a ×63 oil-immersion objective (1.46 NA), and at 5 Hz for 30 s for GCaMP6f using a ×20 objective (0.8 NA). iGluSnFr was imaged live using z-stack acquisitions with a ×63 oil-immersion objective (1.46 NA) in the synaptic chamber. Fixed immunostaining images of SYP/PSD95 were obtained using z-stack acquisitions with a ×63 oil-immersion objective (1.4 NA) using an inverted confocal microscope (LSM 710, Zeiss) coupled to an Airyscan detector to improve signal-to-noise ratio and spatial resolution.

### Synaptophysin/PSD95 analysis

Neurons were fixed by filling the microchambers with PFA/Sucrose (4%/4% in PBS) for 20 min at room temperature (RT) followed by PBS washes and were incubated for 1 h at RT with a blocking solution (BSA 1%, normal goat serum 2%, Triton X-100 0.1%). The synaptic compartment was then incubated with Synaptophysin (Abcam, #AB14692, 1:200) and PSD95 (Millipore, #MAB1598, 1:1,000) primary antibodies overnight at 4 °C and appropriate fluorescent secondary antibodies were incubated for 1 h at RT. The immunofluorescence was maintained in PBS for a maximum of one week in the dark at 4 °C before confocal acquisitions. SYP/PSD95 colocalization analyses were performed using ImageJ. Airyscan images were first thresholded to remove non-specific signal and an area of interest of at least 100 μm in length was defined around neurites. The number of synaptophysin spots overlapping, juxtaposed or separated by no more than 2 pixels (130 nm) to PSD95 spots were counted manually. 3 regions of interest per chamber were randomly selected in at least 6 microchambers from 3 independent cultures (n = number of fields).

### iGluSnFR analysis

Live acquisition of iGluSnFR fluorescence was performed after stimulating cortical neurons for 5 min at 37 °C using chemical stimulation (HEPES 25 mM pH 7.5, NaCl 124 mM, KCl 3 mM, CaCl_2_ 2 mM, glucose 10 mM, glycine 200 µM, strychnine 1 µM). iGluSnFR images were filtered by size using the Bandpass Filter plugin in ImageJ and were then binarized to remove non-specific signals. A region of interest of at least 50 μm in length was defined around neurites using transillumination. The number of spots was then counted using the Analyze Particle plugin. 4 fields per chamber from at least 6 microchambers from 3 independent cultures were analyzed (n = number of fields).

### GCaMP6f analysis

Single cell analysis of calcium fluorescence was performed using Matlab 2014b. Cell bodies detection was achieved manually and ΔF/F traces were calculated using FluoroSNNAP software^37^ on Matlab. Calcium event detection was performed with homemade functions as previously described^3^. Synchronized events were determined as the number of events that occur within a 200 ms window from tested event. Acquisition fields were randomly distributed along the striatal chamber and all responding cells in the field were defined as regions of interest. Each condition was tested using 3 fields per chamber (each including at least 20 neurons) from 9 microchambers prepared from 3 independent cultures (n = neurons unless otherwise stated).

### Trafficking analysis

Live-cell video-acquisitions of BDNF-mCh and Mito-DsRed2 were analyzed using kymographs generated using KymoToolBox plugin for ImageJ^10^ with a length of 100 µm (x-axis) and a total time of 30 s (BDNF-mCh) or 5 min (mito-DsRed2) (y-axis) to extract the following kinetics parameters:

$$\begin{array}{cc}{\rm{Anterograde}}\,{\rm{velocity}},\,{\rm{Vma}}\,(\mu {\rm{m}}/{\rm{s}}) & Vma=\frac{Anterograde\,Distance\,(\mu m)}{Time\,(s)}\end{array}$$

$$\begin{array}{cc}{\rm{Retrograde}}\,{\rm{velocity}},\,{\rm{Vmr}}\,(\mu {\rm{m}}/{\rm{s}}) & Vmr=\frac{Retrograde\,Distance\,(\mu m)}{Time\,(s)}\end{array}$$

$$\begin{array}{cc}{\rm{N}}{\rm{u}}{\rm{m}}{\rm{b}}{\rm{e}}{\rm{r}}\,{\rm{o}}{\rm{f}}\,{\rm{a}}{\rm{n}}{\rm{t}}{\rm{e}}{\rm{r}}{\rm{o}}{\rm{g}}{\rm{r}}{\rm{a}}{\rm{d}}{\rm{e}}\,{\rm{v}}{\rm{e}}{\rm{s}}{\rm{i}}{\rm{c}}{\rm{l}}{\rm{e}}{\rm{s}},\,{\rm{N}}{\rm{a}}\,(/100\,\mu {\rm{m}}) & Na=\frac{na}{Axon\,length\,(\mu {\rm{m}})\,}\times 100\,\,(\mu {\rm{m}})\end{array}$$

$$\begin{array}{cc}{\rm{N}}{\rm{u}}{\rm{m}}{\rm{b}}{\rm{e}}{\rm{r}}\,{\rm{o}}{\rm{f}}\,{\rm{r}}{\rm{e}}{\rm{t}}{\rm{r}}{\rm{o}}{\rm{g}}{\rm{r}}{\rm{a}}{\rm{d}}{\rm{e}}\,{\rm{v}}{\rm{e}}{\rm{s}}{\rm{i}}{\rm{c}}{\rm{l}}{\rm{e}}{\rm{s}},\,{\rm{N}}{\rm{r}}\,(/100\,\mu {\rm{m}}) & Nr=\frac{nr}{Axon\,length\,(\mu {\rm{m}})\,}\times 100\,\,(\mu {\rm{m}})\end{array}$$

$$\begin{array}{cc}{\rm{Linear}}\,{\rm{Flow}}\,{\rm{Rate}},\,{\rm{Q}}\,(\mu {\rm{m}}/{\rm{s}}) & Q=|Vma|\ast na+|Vmr|\ast nr\end{array}$$

$$\begin{array}{cc}{\rm{Net}}\,{\rm{Flux}},\,{\rm{D}}\,(\mu {\rm{m}}/{\rm{s}}) & D=|Vma|\ast na-|Vmr|\ast nr\end{array}$$

Five fields per chamber containing at least 4 axons were analyzed from 6 microchambers prepared from 3 independent cultures (n = number of axons).

### Statistical analyses

Statistical analyses were performed using Prism5 (GraphPad Software). The effect of time on synaptic formation (SYP/PSD95), synaptic transmission (iGluSnFR), network connectivity (GCaMP6f) and trafficking kinetics (BDNF-mCh, Mito-DsRed2) was analyzed using one-way ANOVA followed by a Tukey post hoc test. Correlations studies were also analyzed using one-way ANOVA followed by a Tukey post hoc test. Early and late effects were compared using unpaired two-tailed Student’s t-test. The distributions of motile vesicles (BDNF-mCh) were analyzed using a Pearson’s chi-square test. Results are expressed as mean ± SEM. The criterion for statistical significance was set at *p* < 0.05.

## Electronic supplementary material

Supplementary Information

Movie S1

Movie S2
