# Supplementary material for: Neuronal network maturation differently affects secretory vesicles and mitochondria transport in axons
Source: Sci Rep. 2018 Sep 7;8:13429. doi: 10.1038/s41598-018-31759-x (PMC6128875; doi:10.1038/s41598-018-31759-x)
Supplement: Supplementary file 1 — Supplementary Information [file 41598_2018_31759_MOESM1_ESM.pdf]

SUPPLEMENTARY INFORMATION

Neuronal network maturation differently affects secretory vesicles and mitochondria transport in axons

Eve Moutaux[1,2], Wilhelm Christaller[1,2], Chiara Scaramuzzino[1,2], Aurélie Genoux[1,2], Benoit Charlot[3,4], Maxime Cazorla[1,2†*] and Frédéric Saudou[1,2,5†*]

[1]Grenoble Institut Neuroscience, Univ. Grenoble Alpes, F-38000 Grenoble, France,
[2]INSERM, U1216, F-38000 Grenoble, France,
[3]CNRS UMR5214 Institut d'Electronique et des Systèmes, F-34000 Montpellier, France,
[4]Univ. Montpellier 2, F-34000 Montpellier, France,
[5]CHU Grenoble Alpes, F-38000 Grenoble, France

[†]These authors contributed equally to this work.
*Corresponding authors : maxime.cazorla@inserm.fr (M.C.), frederic.saudou@inserm.fr (F.S.)

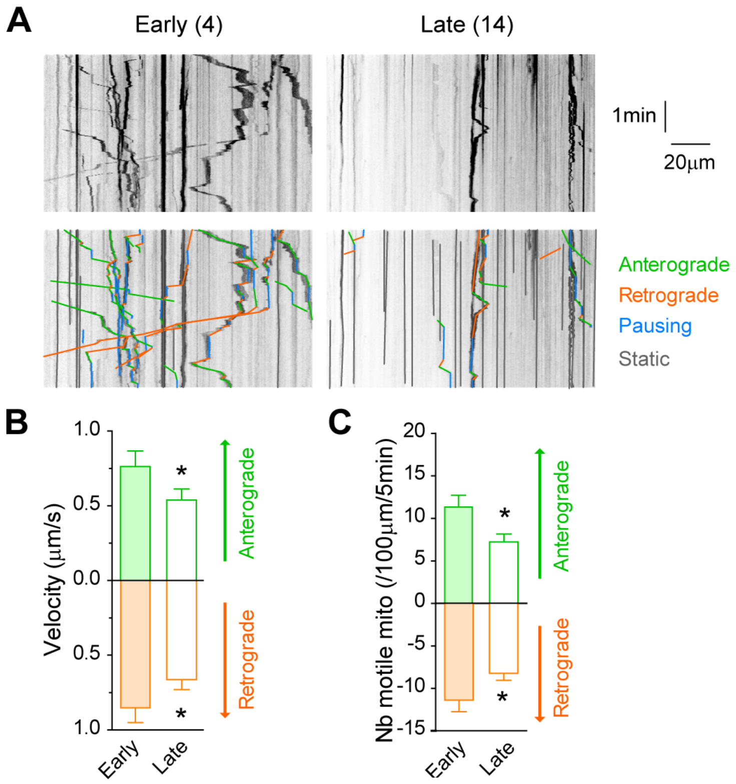

Supplementary Figure S1. Changes in mitochondria axonal trafficking using Mitotracker.

(A) Kymographs at early (DIV 4) and late (DIV 14) stages of network maturation show decreased mitochondria traffic. Anterograde and retrograde movements, pauses and stationary mitochondria are shown. **(B)** Decreased velocity and **(C)** decreased number of motile mitochondria. **(D)** As observed with Mito-DsRed2, the total number of mitochondria is similar between early and late cultures but **(E)** the percentage of stationary units increased. **(F)** These changes translated into a global decrease in linear flow rate but **(G)** with no directional preference. $^*p < 0.05$, $^{**}p < 0.01$, $^{***}p < 0.001$, n = 20.

Supplementary Movie 1. Videorecording of spontaneous neuronal activity using GCaMP6f in an immature corticostriatal network at DIV 7. The video is accelerated 4 times (Scale bar, 100 μm).

Supplementary Movie 2. Videorecording of spontaneous neuronal activity using GCaMP6f in a mature corticostriatal network at DIV 14. The video is accelerated 4 times (Scale bar, 100 μm).
